# The gut microbiome–cardiometabolic axis: insights into obesity, type 2 diabetes, and hypertension

**DOI:** 10.3389/fendo.2026.1948038

**Published:** 2026-08-25

**Authors:** Jhommara Bautista, Ronald Hernández-León, Alexandra Valencia-Valverde, Andrés López-Cortés

**Affiliations:** 1 Cancer Research Group (CRG), Faculty of Medicine, Universidad de Las Américas, Quito, Ecuador; 2 Departamento Cirugia General y Bariátrica, Hospital General San Francisco IEES, Quito, Ecuador

**Keywords:** gut microbiome, hypertension, microbial metabolites, obesity, type 2 diabetes

## Abstract

Alterations in gut microbial ecology have been linked to obesity, type 2 diabetes (T2D), and hypertension, but their biological significance remains difficult to separate from diet, medication use, adiposity, and other host factors. We synthesize evidence on intestinal barrier dysfunction, microbial translocation, low-grade inflammation, and microbiota-derived metabolites as interconnected mechanisms across these disorders. SCFAs, bile acids, trimethylamine N-oxide, tryptophan derivatives, branched-chain amino acid metabolites, and phenylacetylglutamine influence epithelial function, immune activation, insulin signaling, lipid handling, vascular tone, and renal physiology. Cross-cohort comparisons identify the greatest taxonomic overlap between obesity and T2D, whereas hypertension is characterized more consistently by shifts in community structure than by reproducible disease-specific taxa. Dietary modification, prebiotics, probiotics, synbiotics, postbiotics, and fecal microbiota transplantation produce modest and variable benefits, often shaped by baseline microbial features and clinical phenotype. The mechanistic and comparative data position the microbiome as a context-dependent contributor rather than an independent cause of cardiometabolic dysfunction. Progress requires longitudinal cohorts, repeated sampling, integrated multi-omics, standardized protocols, diverse populations, and prospective validation of functional biomarkers and treatment-response signatures before translation into clinical practice.

## Introduction

Cardiometabolic diseases constitute a major cause of premature morbidity, mortality, and healthcare expenditure worldwide. Cardiovascular disease continues to account for the largest share of global deaths, while obesity, type 2 diabetes (T2D), and hypertension contribute substantially to the metabolic and vascular conditions that precede or accompany cardiovascular events. Diabetes illustrates the scale of this burden: approximately 537 million adults were affected in 2021, and the number is projected to reach about 783 million by 2045; more than 90% of cases correspond to T2D. Hypertension further increases the burden through its association with heart failure and renal disease, whereas obesity frequently coexists with impaired glucose regulation, dyslipidemia, and elevated blood pressure. The increasing prevalence of these conditions is associated with demographic, socioeconomic, behavioral, and environmental changes (1–3).

The cardiometabolic continuum describes the progressive accumulation and interaction of metabolic and vascular abnormalities, from excess adiposity and impaired glucose regulation to hypertension and clinically overt cardiovascular disease. Such abnormalities often develop gradually, coexist within the same individual, and confer greater risk than any diagnosis considered in isolation. Their overlap complicates efforts to attribute biological changes to one condition alone. Deeply phenotyped cohorts indicate that many microbiome-related alterations identified in patients with ischemic heart disease are already present in individuals with obesity or T2D who have not developed overt cardiovascular disease. Cardiovascular complications may therefore represent the later expression of a prolonged trajectory that begins with metabolic susceptibility and advances through clustered risk factors and cumulative physiological impairment. Viewing disease as a continuum shifts attention from isolated diagnoses toward the sequence and coexistence of abnormalities that define cardiometabolic progression (4, 5).

The gut microbiome is relevant to cardiometabolic health because microbial composition and functional activity are associated with several clinical and physiological features involved in obesity, T2D, and hypertension. Its structure is influenced by diet, medication exposure, lifestyle, host characteristics, and environmental conditions, which must be considered when interpreting associations with disease. Large population-based studies have related microbiome variation to body composition, glucose regulation, lipid profiles, liver-related measures, and blood pressure. Diet-associated differences have also been linked to favourable and unfavourable cardiometabolic profiles across multinational cohorts. Gut microbiome should therefore be examined within the wider clinical and environmental context of cardiometabolic disease rather than as an independent determinant of metabolic health (6–8).

Interpretation is constrained by the design of much of the available human research. Most studies are cross-sectional and cannot determine whether microbiome differences precede metabolic deterioration, arise as a consequence of disease, or reflect shared exposures. Longitudinal research is expanding, but prospective cohorts with repeated microbial sampling, detailed clinical phenotyping, and long-term follow-up are still uncommon. Confounding is a persistent concern because diet, adiposity, smoking, alcohol use, age, sex, ethnicity, medication exposure, and comorbid disease can influence both microbiome measurements and cardiometabolic outcomes. In advanced disease, polypharmacy and accumulated metabolic abnormalities may obscure associations that were present earlier in the clinical course. Differences in participant recruitment, diagnostic criteria, sequencing methods, analytical pipelines, and covariate adjustment further reduce comparability across studies (9, 10).

Methodological heterogeneity also extends to pre-analytical and laboratory procedures. Stool preservation conditions and DNA extraction protocols can alter the microbial profiles recovered from fecal samples, while differences in sample handling may also affect metabolite measurements. Recent method-comparison studies have demonstrated measurable effects of preservation conditions and DNA isolation procedures on microbial composition and related metabolic readouts. Standardized collection and processing protocols, together with transparent reporting of sequencing and bioinformatic procedures, are therefore necessary for reliable cross-cohort comparison (11, 12).

A separate conceptual challenge arises from the absence of a universally accepted definition of a healthy gut microbiome. Microbial communities vary substantially among individuals, and similar physiological functions may be maintained by different taxonomic configurations. Functional redundancy limits the value of broad compositional metrics when considered in isolation. Many studies continue to underrepresent population diversity, despite indications that microbiome–health associations may differ according to sex, ethnicity, geography, age, diet, and other contextual variables. Large multicohort analyses have improved statistical power and reproducibility, but they cannot fully disentangle microbiome associations from closely related dietary and clinical factors. Research on hypertension has expanded, yet inconsistent microbiome assessment, limited functional profiling, and marked interindividual variability continue to restrict comparison across studies (3, 13, 14).

An integrated analysis of obesity, T2D, and hypertension is needed because the three conditions share clinical risk factors and frequently develop within the same cardiometabolic trajectory. Reviewing them separately can obscure common microbiome-related processes and make it difficult to determine whether reported changes are specific to one disease or reflect broader metabolic impairment. This review examines the role of the gut microbiome in cardiometabolic homeostasis and its involvement in obesity, type 2 diabetes, and hypertension. It integrates evidence on intestinal barrier dysfunction, metabolic endotoxemia, inflammation, microbiota-derived metabolites, and disease-associated microbial alterations; evaluates how these processes converge across the three conditions; and reviews current microbiome-targeted therapeutic strategies. It also identifies priorities for longitudinal, functionally resolved, and clinically representative research.

## Literature search and evidence selection

A narrative literature search was conducted in PubMed up to August, 2026. Search terms included “gut microbiome”, “gut microbiota”, “intestinal microbiome”. “obesity”, “type 2 diabetes”, “insulin resistance”, “hypertension”, and “cardiometabolic disease”, together with terms related to intestinal barrier dysfunction, metabolic endotoxemia, inflammation, microbial metabolites, and microbiome-directed interventions. Reference lists of relevant articles were also examined to identify additional publications.

Recent peer-reviewed literature was prioritized, with particular attention to human cohort studies, longitudinal studies, clinical interventions, systematic reviews, and meta-analyses directly addressing obesity, T2D, hypertension, or related cardiometabolic phenotypes. Earlier studies were retained when they provided relevant mechanistic or foundational evidence. Experimental studies were included when they contributed mechanistic information that could not be established from observational human studies alone. Studies without a direct intestinal microbiome or cardiometabolic context were not considered central to the synthesis.

Interpretation was guided by study design. Cross-sectional findings were treated as associations and were not considered evidence of causality. Longitudinal studies were used to assess temporal relationships, whereas interventional studies and experimental models were used to support mechanistic interpretation when appropriate. Adiposity, glycemic status, diet, and medication exposure were considered relevant sources of confounding, particularly metformin use in studies of T2D. As this was a narrative review, no formal systematic screening, risk-of-bias assessment, or quantitative evidence synthesis was performed. For microbiome-directed interventions, the strength of clinical evidence was qualitatively appraised according to the availability of randomized controlled trials and meta-analyses, consistency across studies, magnitude and durability of reported effects, use of clinically relevant cardiometabolic outcomes, and safety data. Evidence was categorized as moderate, low–moderate, or low. These categories represent a narrative appraisal rather than a formal GRADE assessment, because a systematic risk-of-bias evaluation was not performed.

## The gut microbiome and cardiometabolic homeostasis

The gut microbiome contributes to cardiometabolic homeostasis through the organization of a diverse and functionally coordinated ecosystem. Firmicutes and Bacteroidota generally account for most bacterial abundance, whereas Actinobacteria, Proteobacteria, and Verrucomicrobiota constitute smaller but physiologically relevant fractions. Community composition differs according to age, diet, host genetics, lifestyle, medication exposure, and geography, making a single taxonomic definition of a healthy microbiome difficult. Ecological richness, evenness, functional redundancy, metabolic cross-feeding, and resilience provide a more informative framework. Large metagenomic cohorts further support the existence of several microbiome configurations compatible with favorable host physiology, indicating that functional capacity may be more relevant than broad phylum-level proportions (13, 15, 16). 

A major physiological role of the intestinal microbiota is the processing of substrates that escape digestion in the small intestine. Complex carbohydrates, resistant starches, and selected proteins undergo microbial fermentation and catabolism, generating SCFAs, vitamins, and other bioactive compounds. Acetate, propionate, and butyrate are the main SCFAs produced in the colon. Butyrate serves as an important energy source for colonocytes and sustains epithelial oxidative metabolism, thereby helping preserve the low-oxygen environment required by obligate anaerobes. Acetate and propionate enter the portal circulation and participate in hepatic substrate handling. SCFAs also regulate luminal pH, mucus production, intestinal transit, and enteroendocrine signaling through receptors such as FFAR2 and FFAR3 (6, 17, 18).

Microbial colonization is also involved in the maturation and basal regulation of the mucosal immune system. Commensal antigens contribute to the development of gut-associated lymphoid tissue, secretory IgA responses, intraepithelial lymphocyte populations, and regulatory T-cell differentiation. Selected members of the intestinal microbiota, together with microbiota-derived SCFAs, facilitate regulatory T-cell homeostasis and controlled immune surveillance at the mucosal surface. Microfold cells, tuft cells, dendritic cells, and plasma cells participate in antigen sampling and local immune regulation. This organization enables continuous interaction with resident microorganisms and dietary antigens while maintaining appropriate tolerance and preserving defensive capacity against harmful luminal signals (17, 19).

The intestinal barrier provides the structural basis for stable host–microbiome coexistence. Its outer layer consists primarily of mucins secreted by goblet cells, while the epithelial layer is sealed by occludin, claudins, junctional adhesion molecules, zonula occludens proteins, E-cadherin, and desmosomes. These junctional complexes regulate paracellular transport and limit direct microbial contact with underlying tissues. Microbial metabolites contribute to epithelial energy supply and renewal, whereas specific commensals help maintain mucus production and junctional organization. *Bacteroides vulgatus* and *Bacteroides dorei* have been associated with increased tight-junction expression, while *Akkermansia muciniphila* promotes mucin production and supports colonic mucus-layer thickness. Barrier integrity therefore depends on coordinated microbial, epithelial, and immune functions (6, 20).

Communication between the microbiome and the host occurs through metabolites, modified host molecules, endocrine signals, and neural pathways (21). SCFAs, secondary bile acids, tryptophan derivatives, and cholesterol metabolites act locally or enter the circulation to reach peripheral tissues. Their effects are mediated through metabolite-sensing receptors, nuclear receptors, and specialized transport systems. Enteroendocrine cells provide a hormonal relay by releasing glucagon-like peptide-1, peptide YY, and related peptides in response to luminal metabolic signals. Gut microorganisms also influence serotonin biosynthesis in enterochromaffin cells, creating a route through which intestinal signals reach vagal afferents and central autonomic circuits. Communication pathways link nutrient availability with digestive, endocrine, neural, and cardiovascular regulation (9, 19, 22).

The gut–liver axis is organized around portal transport and enterohepatic circulation. Hepatocytes convert cholesterol into primary bile acids, conjugate them with glycine or taurine, and secrete them into the intestine. Microbial bile salt hydrolases deconjugate these molecules, and additional bacterial enzymes generate secondary bile acids. Most bile acids are reabsorbed in the distal ileum and returned to the liver through the portal vein. Beyond facilitating lipid absorption, bile acids signal through FXR, TGR5, PXR, CAR, and VDR, coordinating lipid handling, glucose metabolism, and energy expenditure. Gut bacteria also participate directly in cholesterol transformation; *Oscillibacter* isolates can generate cholestenone, glycosylated cholesterol, and hydroxycholesterol, potentially modifying the fraction available for host absorption (23–25).

The gut–pancreas axis is mediated mainly through enteroendocrine and bile acid signaling. SCFAs activate FFAR2 and FFAR3 on intestinal L cells, whereas bile acids stimulate TGR5-dependent pathways. Both mechanisms promote glucagon-like peptide-1 secretion, linking microbial metabolism with glucose-dependent insulin release and postprandial nutrient handling. Intestinal FXR–FGF19 signaling adds a further regulatory layer by coordinating hepatic glucose production, bile acid synthesis, and systemic energy use. Pancreatic endocrine activity is therefore embedded within a broader intestinal–hepatic signaling network in which microbial metabolites, gut hormones, and bile acid receptors align nutrient availability with whole-body metabolic demands (6, 24, 26).

The gut–kidney axis extends microbiome-derived signaling to renal physiology. Circulating microbial metabolites interact with renal sensing pathways or undergo host conversion before excretion. SCFAs activate renal G protein-coupled receptors that can influence renin release and vascular regulation, while butyrate-dependent GPR43 signaling has been linked to preservation of renal function in experimental models. Microbial metabolism of tryptophan also generates indole, which is subsequently converted by host enzymes into compounds handled by the kidneys. Through metabolite-mediated, endocrine, and autonomic routes, the kidney participates in a regulatory network connecting intestinal microbial activity with fluid balance, vascular tone, and systemic metabolic homeostasis (3, 27).

## Intestinal barrier dysfunction and metabolic endotoxemia

The intestinal barrier is a selectively permeable interface that separates the luminal microbiota from the internal environment while allowing nutrient and water absorption. Its integrity depends on the mucus layer, a continuous epithelial monolayer, intercellular junctional complexes, and the underlying gut vascular interface. Tight junctions are located at the apical region of adjacent epithelial cells and provide the principal control over paracellular transport. Their transmembrane components include claudins, occludin, and junctional adhesion molecules, which are linked to the actin cytoskeleton through scaffolding proteins such as zonula occludens-1 (ZO-1). This arrangement limits contact between luminal microorganisms and host tissues and restricts the entry of microbial products into the lamina propria and circulation. Coordinated epithelial and vascular control is therefore required to preserve intestinal compartmentalization and cardiometabolic homeostasis (28, 29).

Tight junctions regulate paracellular transport through a size- and charge-selective pore pathway and a less selective leak pathway that permits macromolecular passage when junctional organization is disturbed. Altered claudin composition, reduced occludin abundance, or abnormal localization of junctional proteins can weaken this selectivity. Barrier dysfunction may involve loss of sealing claudins, increased expression of pore-forming claudin-2, and reduced ZO-1 support. Zonulin should be distinguished from zonula occludens proteins: zonulin is a soluble modulator associated with tight-junction disassembly, whereas ZO-1 is an intracellular structural protein. Sustained disruption of these components increases paracellular permeability and produces the phenotype commonly described as “leaky gut”. The term does not imply complete rupture of the intestinal wall, but rather impaired regulation of molecular passage across the epithelium (30, 31).

Dietary and microbial disturbances can alter junctional architecture before cardiometabolic disease is fully established. In experimental models, high-fat and high-sugar feeding reduce intestinal expression of occludin and several claudins, increase the passage of permeability tracers into blood, and raise circulating LPS. Transfer of microbiota from donors with obesity has also produced a thinner mucus layer, widened epithelial junctions, reduced ZO-1, increased claudin-2, and higher serum LPS in recipient mice. Such findings suggest that transferable features of an obesity-associated microbiota may contribute directly to epithelial dysfunction, although results obtained in colorectal carcinogenesis models should be interpreted within their experimental context. Hyperglycemia may further weaken epithelial integrity, supporting a bidirectional relationship in which impaired glucose regulation and intestinal leakage reinforce one another (32, 33).

Once epithelial restriction is weakened, bacterial translocation may involve viable microorganisms, bacterial DNA, membrane fragments, and soluble microbial products (34). Such material can cross the epithelium, enter the lamina propria and mesenteric lymphatic system, or reach the liver through portal venous drainage. The gut vascular barrier provides an additional checkpoint by regulating movement from intestinal tissue into the bloodstream. Concurrent impairment of epithelial and vascular control increases the probability that luminal material will disseminate beyond the intestine. The liver is exposed early because it receives portal effluent directly, whereas bacterial products may subsequently reach adipose tissue and the vascular compartment. Complete dissemination of viable bacteria is not required; repeated entry of comparatively small quantities of microbial material may be sufficient to disturb systemic metabolic regulation (6, 29, 31).

Lipopolysaccharide is the bacterial component most closely associated with this process. LPS is a structural constituent of the outer membrane of Gram-negative bacteria and is normally confined largely to the intestinal lumen. Increased permeability facilitates its passage across epithelial and vascular compartments into portal and systemic circulation. A persistent, modest elevation of circulating gut-derived LPS is termed metabolic endotoxemia, distinguishing it from the substantially greater endotoxin burden associated with acute infection. In experimental obesity, reduced occludin and claudin-1 expression coincides with greater permeability and increased LPS in plasma and adipose tissue. Conversely, preservation of tight-junction expression is accompanied by lower endotoxin exposure and improved insulin sensitivity. The sequence provides a plausible mechanistic link between intestinal damage, obesity-associated insulin resistance, and progression toward T2D (35–37).

A comparable barrier–endotoxemia framework may contribute to hypertension. Spontaneously hypertensive rats exhibit increased proximal-colon permeability, reduced claudin-4 expression, elevated plasma LPS, and impaired endothelium-dependent vasodilation. Restoration of claudin-4 is accompanied by lower intestinal leakage, reduced endotoxemia, and attenuation of blood-pressure elevation, linking intestinal integrity with vascular regulation. Nevertheless, circulating LPS, LPS-binding protein, and zonulin should not be interpreted as interchangeable or direct measures of epithelial permeability. Their concentrations are influenced by intestinal transport, tissue uptake, binding, clearance, and analytical performance, and no single biomarker has achieved complete standardization. Assessment of barrier dysfunction is therefore strengthened by convergent evidence from junctional-protein expression, permeability assays, and microbial translocation. Within this framework, loss of intestinal integrity establishes sustained cardiometabolic exposure to gut-derived bacterial products (30, 38, 39).

## Microbiome-driven inflammation in cardiometabolic disease

Metabolic endotoxemia links microbial sensing with the persistent inflammatory tone associated with cardiometabolic dysfunction. Modest but sustained elevations in circulating lipopolysaccharide (LPS) repeatedly activate innate immune pathways. LPS-binding protein and CD14 facilitate presentation of LPS to Toll-like receptor 4 (TLR4), promoting cytokine production in macrophages and other metabolically responsive cells. Experimental evidence supports a functional role for TLR4-dependent signaling: disruption of bacterial endotoxin production or deletion of host *Tlr4* attenuates obesity, hepatic lipid accumulation, and insulin resistance in susceptible models. Recurrent endotoxin exposure therefore contributes to metaflammation, a chronic low-grade inflammatory state that develops alongside nutrient excess and progressive loss of metabolic control. Unlike a self-limited immune response, metaflammation is maintained by repeated microbial stimulation and metabolic stress, allowing inflammatory mediators to alter tissue function over time (40–42).

TLR4 is a major sensor of endotoxin, although other Toll-like receptors broaden recognition of microbiome-derived products. TLR2 activation can promote dendritic-cell secretion of TNF-α and IL-6, whereas TLR5 recognition of bacterial flagellin can initiate inflammatory responses in resident tissue macrophages. Downstream activation of IκB kinase enables NF-κB nuclear translocation and transcription of cytokines, chemokines, and inflammatory enzymes. NF-κB also cooperates with chromatin regulators. In high-fat-diet models, NF-κB-dependent recruitment of BRD4 to *Ccr2* and *Ccr5* enhancers increases chemokine-receptor expression and macrophage migration. NF-κB–BRD4 cooperation therefore links microbial sensing with sustained recruitment of inflammatory cells rather than a brief local response (37, 43).

NOD-like receptors provide an intracellular layer of bacterial sensing. NOD1 and NOD2 recognize distinct peptidoglycan-derived muropeptides and influence immune-cell activation, antimicrobial responses, and metabolic regulation. Their effects are context dependent rather than uniformly pro-inflammatory. Preserved NOD2 activity can support immune homeostasis, whereas NOD2 deficiency has been associated with CD4+ T-cell imbalance, metaflammation, and more severe insulin resistance in experimental models. Ligand abundance, responding cell type, tissue compartment, and nutritional status determine whether NOD signaling supports physiological regulation or reinforces chronic inflammation. NOD pathways consequently act as modulators of microbiome–immune communication within the broader cardiometabolic inflammatory network, linking intracellular microbial recognition with adaptive immune balance and metabolic control (2, 44).

The NLRP3 inflammasome adds another regulatory checkpoint. Under metabolic overload, mitochondrial dysfunction and reactive oxygen species promote inflammasome activity and favor pro-inflammatory macrophage polarization. Adipose-tissue models further indicate that LPS-induced inflammation depends partly on NF-κB and inflammasome signaling, while NLRP3 silencing reduces inflammatory responses in visceral adipocytes. Increased production of IL-1β and TNF-α by recruited macrophages subsequently amplifies local signaling and extends the metabolic consequences beyond the initiating microbial stimulus. NLRP3 activity is therefore positioned at the intersection of microbial recognition and nutrient-associated cellular stress, where innate immune sensing becomes coupled to adipocyte dysfunction, inflammatory-cell recruitment, and impaired metabolic regulation (41, 45).

TNF-α, IL-1β, and IL-6 are principal mediators connecting innate immune activation with impaired insulin action. TNF-α activates JNK and IKK pathways, promoting inhibitory modification of insulin-receptor substrates and weakening insulin signaling in adipose tissue and skeletal muscle. IL-1β contributes to reduced insulin responsiveness and pancreatic β-cell stress, while LPS-induced IL-6 production participates in systemic inflammatory signaling. Metabolic injury depends less on an isolated cytokine than on persistent exposure to a coordinated inflammatory program. Continued cytokine production increases chemokine expression, recruits additional monocytes, and maintains pro-inflammatory macrophage states. The resulting interaction between immune activation and altered nutrient handling establishes a feed-forward relationship in which inflammation aggravates insulin resistance and insulin-resistant tissues provide additional metabolic signals that sustain inflammation (2, 41).

Immunometabolism helps explain the persistence of the response. Activated macrophages shift from oxidative phosphorylation toward aerobic glycolysis and redirect glycolytic and tricarboxylic-acid-cycle intermediates toward biosynthetic and inflammatory functions. Such metabolic reprogramming supports cytokine synthesis and prolongs inflammatory effector activity under nutrient-rich conditions. Neutrophils and T cells contribute to subsequent tissue remodeling. In experimental models, a transient increase in visceral-adipose-tissue neutrophils precedes expansion of pro-inflammatory Th1 cells, loss of regulatory T cells, and reduced insulin sensitivity; neutrophil depletion prevents the later T-cell shift and preserves insulin responsiveness. Gut intraepithelial lymphocytes provide a complementary example: GLP-1 receptor signaling selectively restrains T-cell-induced, but not LPS-induced, inflammation, indicating that lymphocyte- and endotoxin-driven pathways overlap only partly (46–48).

Inflammation-induced insulin resistance is therefore the cumulative outcome of microbial-product recognition, NF-κB-dependent transcription, NOD-like receptor signaling, NLRP3 activity, cytokine release, and immune-cell reprogramming. Impaired insulin action in adipose tissue promotes lipolysis and greater fatty-acid release, while reduced insulin responsiveness in liver and skeletal muscle disrupts glucose regulation and disposal. Low-grade systemic inflammation may also contribute to blood-pressure dysregulation, although the causal contribution of microbiome-derived endotoxemia to hypertension remains less established than its role in experimental obesity and insulin resistance. Available evidence supports a feed-forward model in which metabolic stress enhances innate immune activation, while inflammation further disrupts glucose and lipid homeostasis across insulin-sensitive tissues (40, 49, 50).

## Microbial metabolites shaping cardiometabolic health

Microbial metabolites provide a functional interface between intestinal microbial activity and host cardiometabolic regulation. Their biological effects depend on substrate availability, microbial enzymatic capacity, host biotransformation, tissue exposure, and receptor distribution. Some compounds act mainly within the intestinal lumen or mucosa, whereas others enter the portal circulation and influence hepatic, endocrine, vascular, and cardiac pathways. A functional perspective is therefore required alongside taxonomic profiling to understand how the gut microbiome contributes to metabolic and cardiovascular homeostasis (51, 52).

SCFAs, primarily acetate, propionate, and butyrate, arise from microbial fermentation of complex carbohydrates and other substrates that reach the colon. Butyrate is preferentially consumed by colonocytes, supports epithelial energy metabolism, and promotes β-oxidation through PPARγ. SCFAs also inhibit histone deacetylases, thereby modifying transcriptional programs involved in barrier integrity, inflammatory control, and substrate handling. Acetate has greater systemic availability, whereas propionate and butyrate undergo substantial intestinal and hepatic extraction before reaching peripheral tissues (53, 54).

SCFA activity is mediated largely through GPR41 and GPR43, also termed FFAR3 and FFAR2. These receptors are expressed in enteroendocrine, immune, adipose, renal, and vascular compartments. Their activation contributes to blood pressure control, limits inflammatory signaling, and supports epithelial integrity. In colonic L cells, butyrate and propionate stimulate glucagon-like peptide-1 (GLP-1) and peptide YY (PYY) release. GLP-1 enhances glucose-dependent insulin secretion, suppresses glucagon, and reduces hepatic glucose production, whereas PYY regulates appetite and gastrointestinal transit. Microbial fermentation can therefore affect insulin sensitivity, energy intake, and vascular function through coordinated receptor and endocrine pathways (55–57).

Bile acids form another major route of microbiome–host communication. Primary bile acids synthesized from cholesterol in the liver are conjugated before secretion into the intestine. Microbial bile salt hydrolases deconjugate bile acids, whereas subsequent dehydroxylation, oxidation, and epimerization generate secondary bile acids such as deoxycholic and lithocholic acids. Microbial biotransformation reshapes the enterohepatic bile acid pool and alters receptor activity, with consequences for lipid absorption, hepatic glucose handling, and systemic metabolic regulation (53, 58, 59).

Farnesoid X receptor (FXR) and TGR5 are the principal metabolic receptors influenced by microbiota-modified bile acids. Intestinal FXR regulates the FGF15/19 axis, which feeds back on hepatic bile acid synthesis and affects gluconeogenesis, glycogen turnover, and lipid handling. TGR5 activation increases intracellular cAMP and promotes GLP-1 secretion from enteroendocrine cells. In adipose, immune, and cardiovascular tissues, TGR5 also influences energy expenditure and inflammatory tone. The physiological consequences of the bile acid pool depend on the relative abundance and receptor affinity of individual species rather than on total concentration alone (54, 58, 60).

Trimethylamine N-oxide (TMAO) is generated through sequential microbial and hepatic metabolism. Gut microorganisms convert choline, phosphatidylcholine, betaine, and L-carnitine into trimethylamine, which is oxidized in the liver by flavin-containing monooxygenases. Experimental work indicates that TMAO can disturb cholesterol homeostasis by increasing HMG-CoA reductase activity and reducing ABCA1- and ABCG1-mediated efflux. It can also promote endothelial activation, platelet responsiveness, coagulation, myocardial fibrosis, and impaired contractility. In metabolically active tissues, TMAO may attenuate insulin signaling and reduce hepatic glycogen storage, although the magnitude and direction of these effects remain context-dependent (61, 62).

Tryptophan-derived indoles have a distinct regulatory profile. Indole-3-propionic acid, indoleacrylic acid, and related metabolites can signal through the aryl hydrocarbon receptor and pregnane X receptor, strengthening tight-junction integrity and supporting IL-22-dependent mucosal regulation. Other tryptophan metabolites engage GPR35- and AMPK-linked pathways, while indole-3-propionic acid has been connected to antioxidant activity, cholesterol efflux, and NAD+ salvage. By preserving epithelial function and limiting oxidative and inflammatory interference with insulin pathways, indoles may contribute to metabolic and cardiovascular stability (63, 64).

Amino acid-derived metabolites can disrupt cardiometabolic signaling when their production or processing becomes imbalanced. Elevated branched-chain amino acid availability or impaired microbial degradation can promote the accumulation of BCAAs and related catabolites, sustain mTORC1 activity, and induce mitochondrial stress, thereby weakening insulin action. Imidazole propionate, generated from histidine, activates p38γ-dependent p62–mTORC1–S6K1 signaling and alters AKT–AMPK regulation. It can also act through the imidazoline-1 receptor in myeloid cells, linking microbial amino acid metabolism with vascular inflammation (65, 66).

Phenylacetylglutamine (PAGln) is generated through sequential microbial and host metabolism. Gut bacteria convert phenylalanine into phenylpyruvate and subsequently into phenylacetic acid through pathways involving phenylpyruvate:ferredoxin oxidoreductase or phenylpyruvate decarboxylase. After absorption, phenylacetic acid is conjugated with glutamine by host enzymes, primarily in the liver and kidneys, to form PAGln. Once in the circulation, PAGln modulates adrenergic signaling and acts as a negative allosteric modulator of the β2-adrenergic receptor. Reduced β2-adrenergic receptor responsiveness during sympathetic stimulation may alter cardiomyocyte contractility, whereas its effects on platelet reactivity appear to involve broader adrenergic receptor signaling (51, 67). The principal microbiome-related functional alterations and their cardiometabolic consequences are summarized in **Table 1**.

**Table 1:** Major microbiome-related functional alterations and their cardiometabolic consequences.

Microbiome-related alteration	Principal host pathway or target	Major cardiometabolic consequence	Main clinical relevance	References
Reduced SCFA-producing capacity or altered SCFA signaling	Reduced butyrate-dependent epithelial support; altered FFAR2/FFAR3 signaling; impaired GLP-1 and PYY release; reduced anti-inflammatory signaling	Impaired intestinal barrier function, altered appetite regulation, reduced insulin sensitivity, and disturbed vascular and blood-pressure regulation	Obesity, T2D, hypertension	(54, 55, 57)
Altered microbial bile acid biotransformation	Changes in secondary bile acid composition and FXR–FGF19/TGR5 signaling	Altered hepatic glucose production, glycogen turnover, lipid handling, GLP-1 secretion, energy expenditure, and inflammatory tone	Obesity, T2D, cardiometabolic dysfunction	(9, 24, 88)
Increased activity of the TMA–TMAO pathway	Altered cholesterol handling, endothelial activation, platelet signaling, coagulation, oxidative and inflammatory pathways	Endothelial dysfunction, enhanced thrombogenicity, impaired cholesterol homeostasis, vascular injury, and myocardial remodeling	Cardiovascular complications and metabolic dysfunction	(61, 62, 122)
Altered production of tryptophan-derived indoles	AhR, PXR and related metabolite-sensing pathways; epithelial and immune signaling	Altered intestinal barrier integrity, inflammatory and oxidative regulation, insulin sensitivity, and cardiovascular homeostasis	T2D and broader metabolic and cardiovascular dysfunction	(51, 54, 63)
Increased BCAA availability or altered microbial BCAA metabolism	Increased microbial biosynthetic potential and reduced BCAA uptake/catabolism; mTORC1-linked impairment of insulin signaling	Elevated circulating BCAA availability and impaired insulin responsiveness	Insulin resistance, obesity, T2D	(51, 54, 84)
Increased imidazole propionate signaling	p38γ–p62–mTORC1–S6K1/insulin-signaling pathways and imidazoline-1 receptor signaling in myeloid cells	Impaired metabolic signaling, systemic/vascular inflammation, atherosclerotic progression, and adverse cardiovascular phenotype	T2D, atherosclerosis, cardiovascular disease	(54, 65, 66)
Altered phenylalanine metabolism and increased PAGln signaling	α- and β-adrenergic receptor signaling, including negative allosteric modulation of β2-adrenergic receptors	Increased platelet responsiveness and thrombosis-related signaling; altered cardiomyocyte β2-adrenergic responsiveness	Cardiovascular disease and heart failure	(51, 67)
Barrier dysfunction and increased translocation of microbial products	Increased circulating LPS; LBP–CD14–TLR4/NF-κB signaling; sustained inflammatory activation	Metabolic endotoxemia, chronic low-grade inflammation, impaired insulin signaling, endothelial dysfunction, and blood-pressure dysregulation	Obesity, insulin resistance, T2D, hypertension	(31, 37, 38)

## Gut microbiome alterations in obesity, type 2 diabetes, and hypertension

Human cohort studies have linked obesity, T2D, and hypertension to differences in gut microbial composition and function, although the frequent coexistence of these conditions complicates attribution of individual microbial features to a specific phenotype. Obesity-related findings are considered mainly in relation to body mass index, total or regional adiposity, and obesity status, whereas T2D-related findings are interpreted in relation to glycemic status, insulin resistance, adiposity, and glucose-lowering medication. Studies adjusting for BMI or adiposity, accounting for metformin exposure, or comparing individuals with similar adiposity but different glycemic status are particularly informative when assessing T2D-associated microbial features. Hypertension-associated findings are considered separately in relation to blood-pressure phenotypes. Taxonomic findings derived from observational studies are interpreted as associations, while causal or mechanistic inferences are restricted to longitudinal, interventional, or experimental evidence when appropriate (68–70).

**Obesity.** Obesity is frequently associated with reduced gut microbial richness and altered abundances of taxa linked to metabolic homeostasis, although lower alpha diversity is not consistently observed across populations. Phylum-level findings have also varied considerably. The Firmicutes-to-Bacteroidetes ratio, initially proposed as a microbial marker of obesity, has been reported as increased, decreased, or unchanged across human cohorts and lacks sufficient reproducibility for disease classification. Among phylum-level alterations, enrichment of Proteobacteria represents one of the more recurrent findings, whereas *Faecalibacterium*, *Akkermansia*, and *Alistipes* are frequently less abundant in individuals with obesity. Lower abundances of *Bifidobacterium*, *Roseburia*, and members of the family Ruminococcaceae have also been described, together with enrichment of *Escherichia–Shigella*, *Fusobacterium*, *Megamonas*, and *Blautia* in some cohorts. Associations involving *Prevotella* and *Ruminococcus* differ between Western and Eastern populations, while enrichment of *Lactobacillus* has been reported more frequently in Western cohorts. Because insulin resistance and impaired glucose regulation frequently coexist with obesity, reported microbial associations cannot always be attributed to adiposity alone. Geographic, clinical, and cohort-dependent variation therefore supports community-level differences associated with obesity but does not establish a universal taxonomic signature (7, 71, 72).

**Type 2 Diabetes.** Interpretation of T2D-associated microbiome findings requires consideration of adiposity and glucose-lowering medication, both of which may contribute to microbial variation. T2D has been associated with genus- and species-level differences in the gut microbiome, although substantial heterogeneity exists across populations. Differences in beta diversity have been reported more consistently than changes in alpha diversity, which vary in direction and magnitude. *Escherichia–Shigella*, *Enterococcus*, *Lactobacillus*, *Fusobacterium*, and *Subdoligranulum* have been reported as enriched in individuals with T2D, whereas *Akkermansia*, *Bifidobacterium*, *Bacteroides*, *Roseburia*, *Faecalibacterium*, and *Prevotella* have frequently been reported as depleted. No taxon, however, shows a consistent association across all cohorts. At higher taxonomic resolution, an analysis of 8,117 metagenomes from ten populations identified 19 species associated with T2D after adjustment for age, sex, BMI, and metformin exposure. Among the associations reproduced across populations were enrichment of *Clostridium bolteae* and depletion of *Butyrivibrio crossotus*. Phylogenetic variation within *Eubacterium rectale* and *Prevotella copri* further suggests that species-level abundance may obscure strain-specific associations with glycemic status (2, 73, 74).

Strain-resolved analyses provide information that is not captured by species-level abundance alone. In the cross-cohort analysis of 8,117 metagenomes, within-species phylogenetic variation across 27 species contributed to interindividual differences in T2D associations, including variation within *Eubacterium rectale*. Strain-specific gene carriage also involved loci related to horizontal gene transfer and quorum sensing. Within *Prevotella copri*, clade A-dominant strains identified in individuals with T2D more frequently retained pathways and enzymes involved in branched-chain amino acid biosynthesis. At the community level, T2D-associated species were also linked to functional alterations in glucose metabolism. Strains assigned to the same species may therefore differ in metabolic capacity and in their association with glycemic phenotype. Functional interpretation requires integration of strain-resolved metagenomics with measurements of microbial gene expression, proteins, or metabolites when available (74, 75).

Comparisons between individuals with similar adiposity but different glycemic status can help distinguish diabetes-associated microbial variation from changes related to obesity. In a Chinese pilot cohort, participants with obesity and T2D showed a different gut microbial profile from participants with obesity and normal glucose tolerance. Lower relative abundances of *Prevotella_9* and *Sutterella* and higher abundances of *Bacteroides* and *Escherichia–Shigella* were reported in the T2D group. Alpha diversity was also higher in participants with obesity and T2D, indicating that reduced microbial diversity is not a consistent feature of metabolic disease. Alpha-diversity measures therefore need to be interpreted together with taxonomic composition and clinical phenotype. Medication exposure is an additional source of confounding. In individuals with T2D, metformin use has been associated with enrichment of the family Enterobacteriaceae, particularly *Escherichia*, and depletion of *Intestinibacter*. Some microbial differences attributed to T2D may therefore partly reflect metformin exposure rather than glycemic status alone (2, 76).

Diabetes-related complications extend microbiome associations beyond glycemic control. In diabetic kidney disease, altered microbial metabolite profiles, intestinal barrier dysfunction, and inflammatory signaling have been linked to the gut–kidney axis. Distal symmetric polyneuropathy has also been associated with distinct gut microbial profiles. A small randomized trial reported improvement in neuropathy scores after fecal microbiota transplantation without a corresponding improvement in glycemic control, although the limited sample size precludes firm clinical inference. Cardiovascular complications involve additional pathways relevant to microbial dysregulation, including endotoxemia, endothelial dysfunction, and microbiota-derived metabolites. The evidence remains insufficient to attribute renal, neurological, or cardiovascular complications independently to gut dysbiosis, particularly because diabetes duration, medication exposure, renal function, and diet may affect both microbiome composition and complication severity (77–79).

**Hypertension.** Evidence linking the gut microbiome to hypertension continues to expand, although a reproducible disease-specific taxonomic signature has not been established. Blood pressure has been associated with microbial diversity, community composition, and the abundance of specific taxa, but the direction and magnitude of these relationships vary across populations. Among 3,695 participants in the SCAPIS cohort, greater alpha diversity was associated with lower variability in 24-hour diastolic blood pressure. Higher abundance of *Streptococcus* was associated with elevated systolic blood pressure, whereas *Intestinimonas massiliensis* and *Dysosmobacter* were associated with lower systolic blood pressure. *Dysosmobacter* was also linked to lower diastolic blood pressure, while ER4 was associated with reduced systolic blood-pressure variability. Additional associations were observed using office blood-pressure measurements; however, the cross-sectional design precluded determination of whether microbial alterations preceded or resulted from changes in blood pressure (80).

Longitudinal evidence remains insufficient to define the temporal relationship between gut microbiome alterations and obesity, T2D, or hypertension. In the Human Phenotype Project, gut microbial functional profiles from 8,859 adults were examined in relation to 38 metabolic health measures, but the reported associations were cross-sectional (7). The prospective multi-ethnic HELIUS cohort extends temporal evidence: baseline fecal microbiota from 4,792 participants was examined in relation to incident cardiometabolic outcomes during follow-up. Because microbiome profiling was performed only at baseline, the study could evaluate prospective associations but not within-person microbial trajectories (10). In T2D, impaired microbial rhythmicity and loss of diurnal oscillation in specific taxa have been reported in individuals with established disease. Available serial data do not clarify whether such alterations precede metabolic dysfunction, develop during disease progression, or arise in response to medication and lifestyle changes. Comparable longitudinal evidence in hypertension is particularly limited. Longitudinal studies incorporating repeated microbiome sampling are therefore needed to distinguish persistent disease-associated alterations from short-term fluctuations driven by diet, bowel habits, circadian rhythms, medication use, and other time-varying exposures (7, 19, 81).

**Cross-condition Overlap and Disease Specificity.** The clearest overlap in reported taxonomic patterns occurs between obesity and T2D, particularly when excess adiposity coexists with impaired glucose regulation. Recurrent observations include lower abundances of *Faecalibacterium*, *Akkermansia*, *Bifidobacterium*, and *Roseburia*, together with enrichment of *Escherichia–Shigella* and other Proteobacteria-associated taxa. In hypertension, overlap with obesity and T2D is more evident at the level of altered diversity and community organization than through a reproducible set of shared taxa. Higher-resolution analyses also reveal condition-dependent signals, including *Clostridium bolteae*, *Butyrivibrio crossotus*, and strain variation within *Eubacterium rectale* in T2D; *Prevotella_9* and *Sutterella* according to glycemic status in obesity; and *Intestinimonas*, *Dysosmobacter*, and a specific *Streptococcus* strain in relation to ambulatory blood-pressure traits. Human evidence therefore supports a pattern of partially overlapping microbial ecological disruption accompanied by condition-specific, population-dependent, and strain-level variation (74, 76, 80). Representative ecological and taxonomic alterations reported across obesity, T2D, and hypertension are summarized in **Table 2**, highlighting both recurrent cross-condition patterns and disease-specific associations.

**Table 2:** Representative gut microbiome alterations across obesity, type 2 diabetes, and hypertension.

Cardiometabolic condition	Community-level pattern	Representative taxonomic associations	Cardiometabolic relevance and interpretation	References
Obesity	Reduced microbial richness is frequently reported, although not consistently across populations. Community composition shows substantial geographic and cohort-dependent variation.	Higher: Proteobacteria; *Escherichia–Shigella*, *Fusobacterium*, *Megamonas*, and *Blautia* in selected cohorts. Lower: *Faecalibacterium*, *Akkermansia*, *Alistipes*, *Bifidobacterium*, *Roseburia*, and Ruminococcaceae.	Associations occur with increased adiposity and impaired glucose regulation, but no individual taxon or phylum-level ratio provides a reproducible microbial signature across populations.	(7, 71, 72)
Type 2 diabetes	Differences in beta diversity are reported more consistently than changes in alpha diversity. Functional and strain-level variation has also been described.	Higher: *Escherichia–Shigella*, *Enterococcus*, *Lactobacillus*, *Fusobacterium*, *Subdoligranulum*, and *Clostridium bolteae*. Lower: *Akkermansia*, *Bifidobacterium*, *Bacteroides*, *Roseburia*, *Faecalibacterium*, *Prevotella*, and *Butyrivibrio crossotus*.	Species- and strain-level associations appear more reproducible than broad diversity changes. Adiposity and glucose-lowering medication, particularly metformin, remain important sources of confounding.	(73, 74, 76)
Hypertension	Altered microbial diversity and community composition have been associated with ambulatory blood-pressure traits, but a reproducible hypertension-specific microbial signature has not been established.	Positive association: higher *Streptococcus* abundance with higher systolic blood pressure. Inverse association: *Intestinimonas massiliensis* and *Dysosmobacter* with systolic blood pressure; *Dysosmobacter* also with diastolic blood pressure.	Evidence is more consistent for community-level and blood-pressure-related microbial patterns than for a conserved disease-specific taxonomic signature.	(3, 50, 80)
Cross-condition pattern	Taxonomic overlap is greatest between obesity and T2D, whereas overlap with hypertension is more evident at the level of community structure.	Obesity and T2D recurrently show lower *Faecalibacterium*, *Akkermansia*, *Bifidobacterium*, and *Roseburia*, together with higher *Escherichia–Shigella* or other Proteobacteria-associated taxa. No taxon is consistently altered across all three conditions.	Findings support partially overlapping, population-dependent ecological disturbances rather than a single conserved cardiometabolic dysbiosis signature.	(73, 74, 80)

## Mechanistic integration of the gut microbiome in cardiometabolic disease

**Figure 1:**
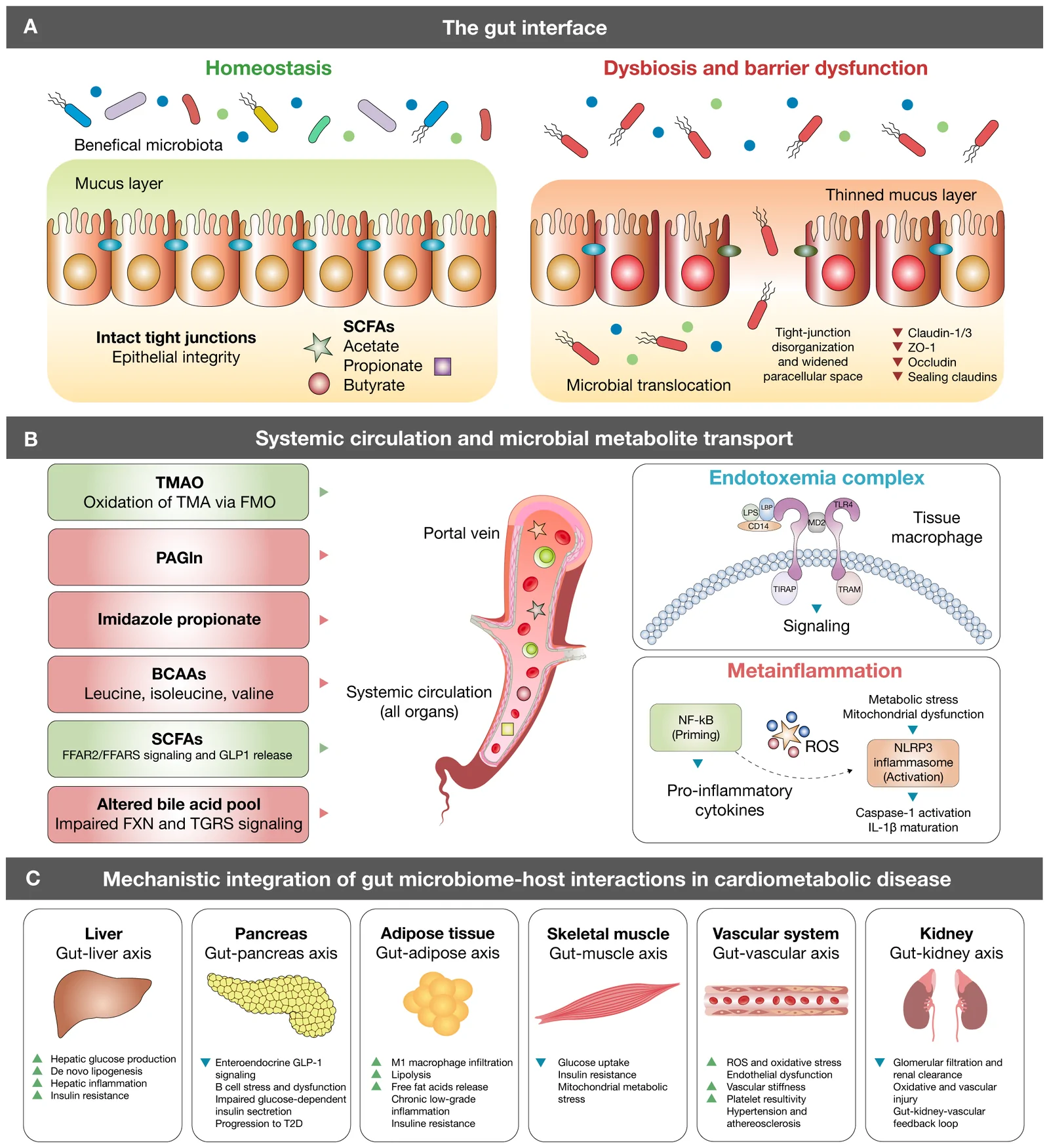
Mechanistic framework linking gut microbial dysbiosis with obesity, type 2 diabetes, and hypertension. **(A)** Intestinal homeostasis is supported by beneficial commensals, SCFA production, mucus integrity, and preserved tight-junction organization. Dysbiosis promotes mucus-layer thinning, increased claudin-2, reduced ZO-1, occludin, and sealing claudins, and microbial translocation. **(B)** Microbial products and metabolites enter the portal and systemic circulation. LPS recognition through the LBP–CD14–TLR4 complex activates NF-κB signaling, while metabolic stress, mitochondrial dysfunction, and ROS promote NLRP3 inflammasome activation and metaflammation. Altered TMAO, PAGln, imidazole propionate, BCAA, SCFA, and bile acid signaling further disrupts metabolic and vascular regulation. **(C)** These pathways converge across the gut–liver, gut–pancreas, gut–adipose, gut–muscle, gut–vascular, and gut–kidney axes, contributing to insulin resistance, β-cell dysfunction, hepatic steatosis, endothelial injury, vascular remodeling, hypertension, and renal impairment. Green arrows indicate protective pathways, whereas red arrows indicate pathogenic pathways.

Cardiometabolic disease arises from interconnected disturbances in intestinal ecology, metabolic regulation, immune activity, and vascular homeostasis. Dysbiosis may occur early in susceptible individuals, although its position within the disease trajectory is not fixed. Diet, medication use, aging, host genetics, and lifestyle can reshape microbial composition and function, whereas obesity, hyperglycemia, altered intestinal transit, and disrupted bile acid metabolism can further modify the gut environment. Dysbiosis may therefore contribute to disease development while also emerging as a consequence of an altered host state. Reciprocal microbiome–host interactions can subsequently sustain metabolic dysfunction across obesity, T2D, hypertension, and cardiovascular disease (5, 82, 83) (**Figure 1**).

Intestinal barrier dysfunction provides a route through which localized microbial disturbances extend beyond the gut. Reduced epithelial integrity increases the translocation of lipopolysaccharide and other microbial products into the portal and peripheral circulation. Persistent exposure promotes metabolic endotoxemia and chronic low-grade inflammation without overt infection. Inflammatory signaling can impair insulin action in the liver, skeletal muscle, and adipose tissue, while disrupted glucose and lipid metabolism can further destabilize microbial function. A reinforcing cycle may consequently develop in which altered microbial states are associated with impaired barrier function and endotoxemia, while experimental evidence supports pathways through which microbial products can promote inflammation and impair insulin signaling. Metabolic deterioration may, in turn, further modify the intestinal environment (84, 85).

Disrupted microbial metabolite signaling provides an additional link between intestinal dysbiosis and systemic disease. The relevant disturbance is not restricted to a single metabolite but involves broader alterations in the microbial processing of carbohydrates, amino acids, bile acids, cholesterol, and lipid-derived substrates. Such changes modify the circulating chemical environment reaching metabolic, renal, and vascular tissues. Altered bile acid signaling can disrupt glucose and lipid regulation, whereas amino acid-derived metabolites may influence insulin sensitivity, appetite regulation, renal burden, platelet activity, and vascular function. Changes in microbial cholesterol transformation may also contribute to interindividual variation in circulating lipid profiles. The gut microbiome may therefore act as an interface between environmental exposure and host metabolism, through which dietary and host-derived substrates are converted into signals associated with cardiometabolic regulation (25, 86).

Once barrier dysfunction and altered metabolite production are established, multi-organ crosstalk can propagate metabolic impairment. The liver receives microbial molecules and co-metabolites directly through the portal circulation, making it particularly susceptible to sustained intestinal disruption. Abnormal inflammatory and metabolic exposure can impair hepatic insulin signaling, increase gluconeogenesis and lipogenesis, alter lipoprotein metabolism, and promote fat accumulation. Pancreatic function may be affected within the same metabolic environment through impaired enteroendocrine signaling, altered glucose-dependent insulin responses, and persistent inflammatory exposure. Hyperglycemia and hepatic metabolic dysfunction can, in turn, modify the intestinal environment, establishing bidirectional interactions between microbial disruption and impaired glucose regulation. Functional studies support an integrated model because bacterial pathways are frequently associated with glucose traits, liver measurements, body composition, and other metabolic outcomes rather than with a single phenotype (7, 87, 88).

The gut–adipose and gut–vascular axes connect altered energy storage with circulatory injury. In adipose tissue, impaired insulin signaling disrupts lipid storage and mobilization, increasing fatty acid release and inflammatory activity with downstream effects on the liver, skeletal muscle, and vasculature. Visceral adipose expansion further reinforces systemic inflammation and metabolic dysfunction. Within the vascular compartment, metabolic endotoxemia, dyslipidemia, oxidative stress, and microbiome-derived metabolites converge on endothelial regulation. Reduced nitric oxide bioavailability, increased vascular tone, monocyte adhesion, platelet activation, and impaired endothelial repair provide mechanistic links to hypertension and atherosclerotic progression. Insulin resistance and endothelial dysfunction can reinforce one another by intensifying inflammation, tissue hypoperfusion, lipid deposition, and target-organ injury (89).

Heart failure with preserved ejection fraction (HFpEF) represents a relevant cardiac phenotype within the cardiometabolic continuum. A small case-control study identified altered gut microbial composition in patients with HFpEF, including lower abundances of *Butyricicoccus*, *Lachnospira*, and *Ruminiclostridium* and enrichment of *Enterococcus*, although the limited sample size precludes definition of an HFpEF-specific microbial signature. Intestinal barrier impairment may provide an additional link. Patients with HFpEF have shown reduced intestinal occludin and claudin-1 expression, higher circulating endotoxin, and increased inflammatory cytokines, supporting an association between impaired intestinal integrity and systemic inflammation. Microbial metabolites may also participate in gut–heart signaling. Indole-3-propionic acid was reduced in human HFpEF cohorts, whereas supplementation attenuated diastolic dysfunction, oxidative stress, inflammation, and intestinal barrier injury in an experimental HFpEF model. Gut dysbiosis, barrier dysfunction, and altered microbial metabolism may therefore contribute to inflammatory and myocardial abnormalities associated with HFpEF, but current human evidence remains insufficient to establish causality or a reproducible HFpEF-specific microbiome signature (90–92).

Renal dysfunction provides a further mechanism of systemic amplification. Reduced filtration promotes the accumulation of gut-derived uremic compounds, including indole- and phenol-related metabolites associated with oxidative stress, vascular injury, thrombosis, cardiac dysfunction, and progressive renal decline. Hypertension, diabetes, and vascular disease can simultaneously reduce renal perfusion and filtration capacity, establishing a gut–kidney–vascular feedback loop. Circulating concentrations of microbial–host co-metabolites may therefore increase even without a proportional rise in intestinal production. Cardiometabolic exposure is determined not only by microbial metabolite generation but also by hepatic transformation and renal clearance, with impaired kidney function prolonging the systemic persistence of potentially harmful compounds (93, 94).

Cardiometabolic progression reflects the cumulative interaction between intestinal dysfunction and organ-specific susceptibility rather than a fixed sequence of events. Obesity and early insulin resistance may create conditions in which dysbiosis, barrier impairment, and altered metabolite production persist. Hepatic glucose overproduction, adipose dysfunction, and impaired pancreatic control can contribute to T2D, whereas vascular and renal injury can promote hypertension and endothelial dysfunction. Over time, dyslipidemia, inflammation, thrombogenicity, and target-organ damage increase the likelihood of overt cardiovascular disease. Prospective studies have associated baseline microbiome features with incident diabetes, dyslipidemia, hypertension, and cardiovascular events, although effect sizes and microbial signatures vary across populations. Diet, medication use, ethnicity, sex, disease severity, and reverse causality remain important sources of heterogeneity. The microbiome is therefore best considered a context-dependent contributor and amplifier within a multi-organ cardiometabolic network rather than an isolated cause of disease (10, 95).

## Microbiome-based therapeutic strategies for cardiometabolic disease

The gut microbiome has emerged as a therapeutic target in obesity, T2D, hypertension, and metabolic syndrome because its composition and functional activity can be modified through dietary, microbial, and community-level interventions. Current strategies include dietary modification, prebiotics, probiotics, synbiotics, postbiotics, fecal microbiota transplantation (FMT), and individualized treatment models. Evidence is strongest for obesity, T2D, and metabolic syndrome, whereas clinical data for hypertension remain more limited. Across cardiometabolic populations, therapeutic effects are generally modest and heterogeneous. In metabolic syndrome, pooled trials have reported improvements in fasting glucose and insulin, but not consistent changes in HbA1c or insulin-resistance indices. Responses vary according to baseline body mass index, intervention type, dose, and duration (39, 96, 97).

Dietary modification remains the most accessible approach to microbiome-directed care. Mediterranean-style and plant-rich diets emphasize fermentable carbohydrates while limiting refined foods and saturated fat. Dietary fiber should not be considered a uniform exposure because its structure, fermentability, food matrix, and habitual intake influence microbial utilization. Resistant starch, inulin-type fructans, arabinoxylans, legumes, whole grains, fruits, and vegetables can alter microbial community structure and metabolite production, although comparable interventions do not consistently produce the same taxonomic changes or degree of metabolic benefit. Dietary intervention therefore represents a practical but context-dependent strategy (53, 98).

Prebiotics are substrates selectively utilized by resident microorganisms to confer a health benefit. Inulin, fructooligosaccharides, galactooligosaccharides, resistant starches, and arabinoxylans are frequently investigated in cardiometabolic populations. Inulin-type fructans have been associated with small reductions in fasting glucose, HbA1c, fasting insulin, and insulin resistance in prediabetes and T2D. Responsiveness may partly depend on the presence of organisms capable of metabolizing the administered substrate. Pretreatment enterotype and the *Prevotella*-to-*Bacteroides* ratio have also been associated with differential responses to selected fibers, supporting microbiome-informed substrate selection rather than uniform supplementation (6, 99).

Probiotics introduce live microorganisms intended to confer a measurable host benefit. Most trials have evaluated *Lactobacillus*, *Bifidobacterium*, or multispecies formulations, with small and inconsistent effects on glycemic control, lipid measures, and adiposity. Product-level interpretation is essential because outcomes depend on strain, dose, formulation, treatment duration, and recipient phenotype. Synbiotics combine live microorganisms with substrates intended to support their activity. A formulation containing inulin, *Akkermansia muciniphila*, *Bifidobacterium infantis*, and butyrate-producing bacteria improved postprandial glucose regulation and increased the abundance of selected administered taxa in adults with T2D. Maintenance of microbial viability during manufacturing, storage, and gastrointestinal transit remains a practical constraint (6, 100).

*Akkermansia muciniphila* provides a clinically relevant example of microbiota-guided probiotic administration. In a 12-week randomized, double-blind, placebo-controlled trial involving adults with overweight or obesity and T2D, supplementation with the *A. muciniphila* strain AKK-WST01 produced no significant overall differences in body weight or HbA1c compared with placebo. However, participants with low pretreatment abundance showed efficient colonization and reductions in body weight, total and visceral fat mass, and HbA1c, whereas those with high abundance showed poor colonization and no comparable clinical benefit. Pre-existing microbial conditions may therefore influence whether an administered strain engrafts and produces measurable metabolic effects (53, 101).

Postbiotics comprise preparations of inactivated microorganisms and their cellular components, whereas isolated metabolites such as butyrate are considered microbiome-derived products rather than postbiotics. Human cardiometabolic data remain limited. A recent meta-analysis of randomized trials found reductions in serum insulin, waist circumference, triglycerides, and C-reactive protein, but no consistent improvement across glycemic, anthropometric, or blood-pressure outcomes. Interpretation is further limited by marked differences in microbial source, preparation, dose, treatment duration, and study population. On this basis, the evidence supporting postbiotics for cardiometabolic disease remains low (96, 102, 103).

FMT transfers a complex donor-derived microbial community to a recipient. Trials in obesity and metabolic syndrome have reported short-term changes in insulin sensitivity, fasting insulin, HDL cholesterol, and selected butyrate-producing taxa, but pooled analyses have not demonstrated consistent improvements in body weight, glucose, HbA1c, insulin resistance, or most lipid outcomes. Donor characteristics, administration route, recipient microbiota, dietary intake, inflammatory status, and bile acid profiles may influence engraftment and clinical response. In a phase 2 study, recipient characteristics and donor-strain engraftment were associated with changes in HOMA2-IR. Pathogen transfer and the transmission of undesirable metabolic traits remain relevant safety concerns (104, 105).

The strength of clinical evidence varies across microbiome-directed interventions. Prebiotics have low-to-moderate support for small improvements in glycemic and metabolic surrogate outcomes, although responses differ according to substrate, dose, dietary background, and baseline microbial composition. Probiotics have moderate support for modest short-term improvements in selected glycemic measures in T2D, but evidence for sustained effects on body weight, blood pressure, or cardiovascular outcomes remains limited. Synbiotics have low-to-moderate support, with favorable metabolic effects reported in clinical trials, although differences in formulation and study design make it difficult to determine the contribution of individual components (106). Evidence for postbiotics remains low because human studies are relatively few, preparations are heterogeneous, and long-term cardiometabolic outcomes have rarely been assessed (103). FMT also has low support for cardiometabolic indications. Although donor microbiota engraftment and short-term metabolic changes have been documented, effects on body weight, glycemic control, insulin resistance, and lipid profiles have not been consistent across trials (107). None of these interventions has yet shown consistent, durable effects on clinically relevant cardiometabolic endpoints across adequately powered trials, and their use in obesity, T2D, or hypertension therefore remains investigational.

Antibiotics can modify gut microbial composition, but their use as microbiome-directed therapy in cardiometabolic disease remains uncertain. In diet-induced obese mice, vancomycin and bacitracin improved glucose tolerance and insulin resistance together with increased GLP-1 secretion. Comparable metabolic benefits have not been demonstrated consistently in humans. In men with obesity and prediabetes, vancomycin altered microbial composition and SCFA and bile acid metabolism without improving insulin sensitivity, whereas amoxicillin produced smaller microbiome changes (108, 109). Rifaximin also did not improve endotoxemia, glucose homeostasis, or lipid metabolism in adults with obesity and metabolic syndrome. Broad antibiotic exposure may additionally reduce microbial diversity and increase the intestinal burden of antimicrobial-resistance genes. Clinical evidence therefore does not currently support routine antibiotic use to improve the gut microbiome in obesity, T2D, or hypertension. More selective targeting of defined microbial taxa or functions remains an area for investigation (110, 111).

Pharmacomicrobiomics of Cardiometabolic Therapies and the Gut Microbiome. Pharmamicrobiomics examines bidirectional relationships between drug exposure and the gut microbiome. Medications may alter microbial composition and metabolic activity, while microbial genes and enzymatic functions can influence drug metabolism and treatment response. This relationship is particularly relevant in T2D, where glucose-lowering therapies may contribute to microbial differences otherwise attributed to the disease itself and may also induce treatment-associated functional changes in the intestinal microbiome (112, 113).

Metformin provides the best-characterized example. Metagenomic analyses have shown that metformin exposure accounts for part of the microbial variation previously attributed to T2D, including increased relative abundance of *Escherichia*. In a double-blind trial involving treatment-naive individuals with T2D, four months of metformin produced marked compositional and functional changes in the gut microbiome. Transfer of post-treatment microbiota to germ-free mice improved glucose tolerance, supporting partial microbial mediation of the metabolic response. A separate metagenomic and metabolomic study identified decreased *Bacteroides fragilis*, increased glycoursodeoxycholic acid, and reduced intestinal FXR signaling shortly after metformin initiation (114, 115).

Human evidence for GLP-1 receptor agonists is less consistent. A 12-week randomized placebo-controlled trial found no significant effect of liraglutide on overall intestinal microbiota composition or alpha and beta diversity. In a pilot study of 52 individuals receiving liraglutide or dulaglutide, microbial beta diversity differed between glycemic responders and non-responders, and *Bacteroides dorei*, *Lachnoclostridium* sp., and *Mitsuokella multacida* remained associated with response after adjustment for selected clinical variables. The small sample size and cross-sectional design limit interpretation of these features as predictive biomarkers. More recently, longitudinal sampling during semaglutide treatment identified no changes in microbial diversity, genus abundance, or predicted functional pathways that remained significant after correction for multiple testing. All participants in that study were receiving stable metformin therapy, further limiting attribution of microbial variation specifically to semaglutide. Baseline microbial features showed preliminary associations with treatment response, but current human data do not establish the gut microbiome as a mediator of GLP-1 receptor agonist efficacy (116–118).

Evidence for other cardiometabolic drugs is also heterogeneous. Rosuvastatin caused limited changes in microbial taxonomic composition in a randomized placebo-controlled trial but altered microbial genes involved in choline and betaine metabolism, including pathways related to trimethylamine production. GLP-1 receptor agonists have shown variable effects. Liraglutide did not significantly change overall intestinal microbiota composition during a 12-week randomized trial, whereas longitudinal analysis during semaglutide treatment identified changes in selected microbial taxa and predicted functions without significant changes in alpha or beta diversity. Empagliflozin has been associated with increased abundance of several SCFA-producing taxa and lower abundance of Escherichia–Shigella, Bilophila, and Hungatella in patients with T2D, although another longitudinal study found no significant change in overall microbial diversity or community composition. The contribution of microbiome modification to the clinical effects of statins, GLP-1 receptor agonists, and SGLT2 inhibitors remains unresolved (116, 119, 120). Representative human drug–microbiome interactions relevant to cardiometabolic therapy are summarized in **Table 3**.

**Table 3:** Human evidence on drug–microbiome interactions relevant to cardiometabolic therapy.

Drug or drug class	Human evidence	Main reported microbiome-related findings	Pharmacomicrobiomic interpretation	References
Metformin	Interventional studies in individuals with treatment-naive or newly diagnosed T2D	Metformin induces compositional and functional microbiome changes, including increased *Escherichia*. Reduced *Bacteroides fragilis*, increased glycoursodeoxycholic acid, and attenuation of intestinal FXR signaling have also been reported. Transfer of post-treatment microbiota improved glucose tolerance in germ-free mice.	Metformin provides the strongest evidence of a bidirectional drug–microbiome relationship among glucose-lowering therapies. Experimental transfer studies support partial microbial involvement in its metabolic effects, although the magnitude of this contribution in humans remains uncertain.	(114, 115)
GLP-1 receptor agonists	Randomized, pilot, and longitudinal human studies involving liraglutide, dulaglutide, and semaglutide	Liraglutide did not significantly alter overall microbial composition or alpha and beta diversity in a randomized trial. Microbial differences between glycemic responders and non-responders have been reported in a small pilot study. During semaglutide treatment, nominal taxonomic and functional changes did not remain significant after multiple-testing correction.	Baseline microbial features may be associated with treatment response, but current human evidence does not establish microbiome modification as a mediator of GLP-1 receptor agonist efficacy.	(116–118)
SGLT2 inhibitors	Interventional and longitudinal human studies, primarily involving empagliflozin	Empagliflozin increased several SCFA-producing taxa, including *Roseburia*, *Eubacterium*, and *Faecalibacterium*, and reduced *Escherichia–Shigella*, *Bilophila*, and *Hungatella* in one clinical study. A subsequent longitudinal study detected no significant changes in alpha or beta diversity after correction for multiple testing.	Microbiome and metabolite changes may accompany empagliflozin treatment, but findings are not consistent across cohorts and their contribution to glycemic or cardiovascular benefit remains unresolved.	(118, 119)
Statins	Randomized intervention and large observational human studies	Rosuvastatin produced little change in overall taxonomic composition but reduced microbial genetic capacity related to choline/betaine transport and metabolism and the TMA pathway. In a large cross-sectional cohort, statin use was associated with a lower prevalence of the dysbiosis-associated Bact2 enterotype among participants with obesity.	Human data suggest that statin exposure may be associated with functional and ecological microbiome differences even when broad taxonomic shifts are limited. Evidence that microbiome modification contributes directly to lipid-lowering or cardiovascular efficacy remains insufficient.	(120, 135)

Heterogeneous responses to dietary interventions, probiotic supplementation, and FMT have encouraged the development of precision nutrition and personalized microbiome-based medicine. Individualized programs can integrate habitual diet, postprandial glucose and triglyceride responses, anthropometry, clinical history, physical activity, and microbial composition. In an 18-week randomized trial, a microbiome-informed program reduced triglycerides and improved body weight, waist circumference, HbA1c, and diet quality compared with standard advice, while also altering microbial beta-diversity. LDL cholesterol, blood pressure, fasting glucose, and insulin did not differ significantly between groups. Comparable stratification approaches may support probiotic selection, prebiotic matching, and donor–recipient selection for FMT, although prospective validation and standardized decision rules remain necessary (101, 104, 121),

Emerging therapies seek to modify defined microbial functions rather than replace the entire intestinal community. Rationally designed consortia may provide greater compositional control than donor-derived FMT, while selected next-generation commensals and microbial products may permit narrower functional targeting. Metabolite-directed strategies include inhibition of microbial trimethylamine production to reduce downstream trimethylamine N-oxide formation. Experimental blockade of this pathway has attenuated diet-related cardiovascular injury in preclinical models. Defined microbial consortia, function-specific inhibitors, and standardized microbial products remain investigational therapeutic platforms (96, 122, 123).

## Conclusions and future perspectives

Human studies have consistently associated gut microbial variation with obesity, T2D, and hypertension, although the magnitude and reproducibility of individual microbial associations vary among populations. Recurrent observations include changes in community structure, depletion of several SCFA-producing taxa, impaired intestinal barrier function, and altered microbial metabolic activity. These findings support biological involvement of the gut microbiome in cardiometabolic dysfunction but do not establish clinical utility. No taxonomic signature has been reproduced sufficiently across populations or across the three conditions to support disease classification. Diet, medication exposure, adiposity, age, sex, geography, and metabolic status remain important sources of variation. Functional and metabolic measurements may be more informative than individual taxa, but their clinical relevance requires further validation (40, 60, 124).

Current clinical assessment of obesity, T2D, and hypertension remains based on established anthropometric, metabolic, and cardiovascular measures rather than microbiome-derived markers. Microbiome composition, microbial metabolites, and microbiome-derived signatures have not been validated as diagnostic, prognostic, or treatment-monitoring tools for these conditions. Their performance has not been shown consistently to provide clinically useful information beyond measures such as body mass index, glycated hemoglobin, lipid concentrations, and blood pressure. Most supporting data derive from observational cohorts, experimental studies, or relatively small clinical interventions rather than prospective studies designed to guide patient management (95, 98, 125).

Therapeutic evidence requires the same distinction. Dietary modification is an established component of cardiometabolic management, but its clinical benefit cannot currently be attributed specifically to microbiome modulation. Prebiotics, probiotics, synbiotics, and postbiotics have produced small or inconsistent changes in metabolic outcomes, while FMT has not shown reproducible benefits across major cardiometabolic endpoints. Differences in formulation, dose, treatment duration, baseline microbiota, background diet, and recipient phenotype contribute to variation among studies. Safety and donor-related considerations provide additional limitations for FMT in cardiometabolic indications. These microbiome-directed approaches therefore remain investigational for the treatment of obesity, T2D, and hypertension (3, 60, 126).

Multi-omics signatures, microbiome-derived biomarkers, and microbiome-informed prediction models remain investigational. Further studies should integrate quantitative metagenomics and metatranscriptomics with metabolomics, host transcriptomics, dietary information, medication exposure, and detailed clinical phenotyping. Such integration may help distinguish microbial presence from functional activity and relate community-level processes to measurable host responses (127). Metabolomics is particularly relevant because circulating and fecal metabolites reflect contributions from diet, microbial metabolism, and host processing. Integrated analyses may also help determine which functional features are shared across cardiometabolic disorders and which are associated with specific clinical phenotypes. Proposed signatures require experimental confirmation and prospective validation before clinical application (75, 128).

Artificial intelligence and machine-learning methods have been applied to high-dimensional microbiome and multi-omics datasets, including models of individual glycemic responses and prediction of long-term T2D remission after bariatric surgery. Their clinical value remains uncertain. External validation, calibration, comparison with conventional clinical models, and transparent reporting of variables contributing to prediction are required before such models can be considered for clinical use. Performance should also be assessed across populations, sequencing platforms, and analytical pipelines to determine whether predictive accuracy is reproducible outside the original study setting (129–131).

Microbiome biomarker development requires similar caution. Candidate taxa, microbial functions, and metabolites should demonstrate reproducibility, temporal stability, and prospective performance in independent cohorts using standardized quantitative methods. Renal function, diet, medication exposure, intestinal transit, and other host factors require consideration because they can modify circulating metabolite concentrations and microbiome–metabolome associations. Multivariable signatures may provide greater predictive information than individual taxa or metabolites, but their value depends on improving prediction beyond routine clinical variables and retaining performance during external validation. Biomarkers proposed for early detection should also be evaluated before overt disease develops and against clinically meaningful outcomes rather than solely by their ability to distinguish cases from controls (128, 132).

Longitudinal and interventional studies are essential for resolving temporal relationships and reducing uncertainty from confounding. Repeated microbiome sampling should be accompanied by contemporaneous assessment of adiposity, glycemic status, diet, physical activity, smoking, alcohol use, intestinal transit, antibiotic exposure, renal function, and cardiometabolic medications. Population diversity also requires explicit consideration because microbiome associations may differ across geographic, ethnic, demographic, and dietary contexts. Cross-cohort analysis of 8,117 metagenomes from ten cohorts in the United States, Europe, Israel, and China identified reproducible T2D-associated species while also revealing substantial strain-level variation. Large-scale analysis of more than 34,000 participants from the United States and United Kingdom similarly identified reproducible diet- and health-associated microbial patterns, with validation in more than 7,800 additional samples. Such findings support external and cross-population validation before microbiome signatures identified in individual cohorts are considered broadly generalizable (10, 13, 74). For glucose-lowering therapies, medication exposure should be considered both a confounder and a biological variable. Pharmacobiomic studies require pretreatment and longitudinal sampling, strain- and function-resolved profiling, and independent validation of microbial features proposed to predict therapeutic response (133).

Clinical implementation will require evidence that microbiome-derived information adds clinically useful information beyond established variables and improves clinically meaningful outcomes. Patient stratification based on microbial functions, metabolite profiles, clinical phenotype, and previous treatment exposure remains under investigation as a means of identifying differential responses to dietary, microbial, pharmacological, or surgical interventions. Microbiome-directed products additionally require reproducible manufacturing, standardized safety monitoring, appropriate regulatory pathways, and consistent reporting standards. Until prospective studies demonstrate clinical utility, microbiome-based biomarkers and microbiome-directed interventions should remain investigational and should not replace established lifestyle and pharmacological management. Research priorities should therefore extend beyond descriptive catalogues of disease-associated taxa toward functionally defined biomarkers and interventions evaluated in independent populations and against clinically relevant endpoints (129, 134).
